# Advances in Research on the Cardiovascular Complications of Acromegaly

**DOI:** 10.3389/fonc.2021.640999

**Published:** 2021-04-02

**Authors:** Han Yang, Huiwen Tan, He Huang, Jianwei Li

**Affiliations:** ^1^ Department of Endocrinology and Metabolism, West China Hospital, Sichuan University, Chengdu, China; ^2^ Department of Endocrinology and Metabolism, Chongqing Sixth People’s Hospital, Chongqing, China; ^3^ Department of Cardiology, West China Hospital, Sichuan University, Chengdu, China

**Keywords:** acromegaly, hypertension, cardiomyopathy, cardiac systolic and diastolic function, echocardiography

## Abstract

Cardiovascular-related complications are one of the most common complications in patients with acromegaly, and can lead to an increased risk of death. Hypertension and cardiomyopathy are the main cardiovascular complications. The characteristics of acromegalic cardiomyopathy are concentric biventricular hypertrophy and diastolic dysfunction. In addition, arrhythmia and heart valve disease are common cardiac complications in acromegaly. Although the underlying pathophysiology has not been fully elucidated, the spontaneous overproduction of GH and IGF-1, increasing age, prolonged duration of disease and the coexistence of other cardiovascular risk factors are crucial to cardiac complications in patients with acromegaly. Early diagnosis and appropriate treatment of acromegaly might be beneficial for the prevention of cardiomyopathy and premature death.

Acromegaly is a chronic neuroendocrine disease with a prevalence of 83-133 cases per one million people (1–7). Approximately 98% are caused by pituitary adenomas that secrete growth hormone (GH) (8, 9), and 2% are caused by pituitary hyperplasia, ectopic growth hormone or ectopic growth hormone releasing hormone (GHRH) secretion. Excessive growth hormone stimulates the liver to produce high levels of insulin-like growth factor-1 (IGF-1). Excessive secretion of GH and IGF-1 has a long-term effect on tissues and multiple organs, which will lead to the excessive proliferation of soft tissues, bones and cartilage of the whole body, causing facial changes, enlarged hands and feet, thick skin, enlarged internal organs, bone and joint disease and obstructive sleep apnea syndrome(OSAS). The quality of life of patients with acromegaly is seriously affected by these disorders. In addition, pituitary tumors can not only cause compression symptoms, but also cause a corresponding increase in the incidence of cardiovascular-related diseases, growth hormone-related metabolic abnormalities, high blood pressure, respiratory diseases, and malignant tumors such as thyroid nodules/thyroid cancer and colon cancer (10), resulting in a shortened life of patients with acromegaly. Compared with the general population, the risk of death in patients with acromegaly had been increased by 61%, and cardiovascular-related complications are the main risk factors affecting the survival rate of patients with acromegaly (11). The common cardiovascular complications in patients with acromegaly mainly include hypertension, acromegalic cardiomyopathy, valvular disease, and arrhythmia (12, 13). The main features of acromegalic cardiomyopathy are ventricular concentric hypertrophy and impaired diastolic function. Autopsy results found that up to 93% of patients with acromegaly had myocardial hypertrophy (14), and up to 60% of patients with acromegaly had arrhythmia, hypertension or valvular heart disease (12). The occurrence of cardiovascular complications may triple the chance of hospitalization for patients with acromegaly and significantly increase the average annual medical expenses (15). More importantly, the presence of any cardiovascular disease at the time of diagnosis of acromegaly indicates a significant increase in the risk of death, and the mortality rate may even reach 100% within 15 years (12). The treatment of acromegaly has been developed in many medical centers, and with the increase in the use of surgery, drugs and stereotactic radiotherapy to enable faster and better clinical and biochemical control, the quality of life and survival of patients with acromegaly has been greatly improved. Recent studies have shown that strict control of GH and IGF-1 levels has reduced the death risk of acromegaly to a level comparable to that of the general population (13, 16–18). However, if GH and IGF-1 remain uncontrolled in patients with acromegaly, systolic dysfunction and even heart failure may eventually occur, which will seriously affect the survival of patients. However, the mechanism of abnormal heart structure and function in acromegaly has not yet been fully elucidated. The present article will review the occurrence, mechanism and related risk factors for common cardiovascular diseases in patients with acromegaly.

## Hypertension

Hypertension is considered one of the most relevant negative prognostic factors for mortality in acromegaly (12, 19). The incidence of hypertension in patients with acromegaly was 11.9%-54.7%, with an average of 33.6% (20). During ACROSTUDY (ACROSTUDY™ is a global non-interventional surveillance study of long-term treatment with pegvisomant initiated in 2004 for evaluating outcomes in patients with acromegaly), 68 patients of acromegaly deaths were reported in the hypertension cohort, while 10 in the cohort without hypertension (19). However, studies have shown that the incidence may be overestimated (21, 22). Minnit et al. observed that, in 40 patients with acromegaly, the incidence of hypertension was 42.5% as assessed by the average of three consecutive random measurements of blood pressure by a standard mercury blood pressure meter, while the incidence of hypertension as assessed by ambulatory blood pressure monitoring (ABPM) was 17.5% (21). A similar result was recently reported by Costenaro et al., with ABPM being assessed at 22.9% in 37 patients with acromegaly, compared with an average rate of hypertension of 32.4% with two random blood pressure tests (22). Therefore, ABPM is more objective and accurate to evaluate the blood pressure, which should be recommended in acromegaly.

Acromegaly-associated hypertension is characterized by special manifestations, with low systolic blood pressure and high diastolic blood pressure (23, 24). Vitale and colleagues found that compared with age- and sex-matched nonacromegalic patients with hypertension, patients with acromegaly had lower systolic blood pressure than nonacromegalic hypertensive controls, while diastolic blood pressure was higher than nonacromegalic hypertension, which suggested that increased vascular resistance may be related to the excessive GH and IGF-1 induced growth of vascular smooth muscle cells (23), and it may also be related to water and sodium retention caused by GH and IGF-1 (24). Additionally, compared with hypertension patients with non-acromegaly, ABPM results showed that approximately 50% of acromegaly hypertension is nondipper hypertension (nocturnal blood pressure drop is less than 10%), which might be related to acromegaly patients’ overreaction to sympathetic nerve stimulation (25).

At present, there are still controversies about the risk factors for hypertension in patients with acromegaly. Studies had found that blood pressure level was positively correlated with IGF-I level (26). Costenaro et al. observed that patients with uncontrolled acromegaly had a higher incidence of hypertension than those with remission of acromegaly and nonacromegalic controls (22). Sardella had pointed out that patients with uncontrolled acromegaly had a 6-fold increase in the risk of hypertension compared with patients who achieved remission after treatment (27). The meta-analysis of Schutte et al. included 3 prospective RCT studies, 14 cross-sectional studies, and 3 case-control studies. A total of 11704 acromegaly subjects and 912 healthy controls were included. The results showed that when the concentration of IGF-1 exceeded 191 ng/ml, blood pressure was significantly positively correlated with IGF-1 (r=0.31, P<0.001), when IGF-1 was lower than 191ng/ml, blood pressure was negatively correlated with IGF-1 (28). This study suggested that in patients with acromegaly who had not yet been completely controlled, blood pressure might be positively correlated with IGF-I level. Therefore, partial or complete remission of acromegaly will be beneficial for blood pressure control. However, in another study, blood pressure was not found to be related to IGF-1 level (23). And patient age and body mass index (BMI) may also be important determinants of hypertension in patients with acromegaly. Sardella and her colleagues observed that the blood pressure of patients with acromegaly was positively correlated with BMI and age (27). However, whether family history of hypertension and sex were related to hypertension in acromegaly is still controversial. A study included 200 patients with acromegaly and 200 subjects with nonacromegaly matched by sex, age, BMI, and smoking habits to assess the prevalence and risk factors for hypertension. And the results suggested that patients with acromegaly have a higher prevalence of hypertension than the control group [hazard ratio (HR) 1.9, P=0.0002], and the risk of hypertension increases with age. And the study also found that 62% of nonacromegalic patients with hypertension had a family history, while only 30% of patients with acromegaly had a family history (23).

The mechanism of hypertension in patients with acromegaly has not yet been fully elucidated (24–29). The occurrence of hypertension may be due to the comprehensive effects of long-term high levels of GH/IGF-1 on the cardiovascular system, kidneys and other organs, which ultimately leads to an increase in extracellular fluid volume and peripheral vascular resistance. Moreover, the accompanying myocardial hypertrophy, OSAS, and insulin resistance in patients with acromegaly can also cause or aggravate hypertension. And the direct or indirect effects of GH/IGF-I cause water and sodium retention. Half a century ago, researchers proposed that GH and IGF-1 could promote water and sodium retention by activating the renin-angiotensin-aldosterone system (RAAS), but there were still controversies. Some studies showed that GH could increase the levels of renin and aldosterone, but other studies found that without relying on the action of plasma renin, excessive GH could directly stimulate the rise in aldosterone levels, leading to hypertension in patients with acromegaly (20). Bielohuby et al. found that aldosterone was increased with chronic GH excess, and the aldosterone level decreased significantly after acromegaly was controlled, but renin concentrations were unaffected, which suggesting that GH might directly promote the secretion of aldosterone (29). The precise mechanism by which chronic excess GH increases aldosterone is unclear, but this study showed that it was unrelated to increased renin, excess IGF-1, or increased adrenal aldosterone synthase expression. Actually, aldosterone synthase is also expressed in some extra-adrenal tissues (30, 31). And excessive growth hormone may affect the synthesis of extra-adrenal aldosterone. GH/IGF-I can also directly act on the sodium channel (epithelial sodium channel, ENaC) of the kidney and renal tubular epithelial cells to increase its activity and cause water and sodium retention (32). There is another mechanism of hypertension in patients with acromegaly, which is increased peripheral vascular resistance. Maison et al. compared 10 patients with acromegaly with normal blood pressure and 10 healthy controls. The results showed that nitric oxide (NO)-mediated vascular endothelium-dependent diastolic function was impaired in patients with acromegaly (33). Nitric oxide is a powerful vasodilator that can also reduce platelet aggregation and activation, inhibit the proliferation and migration of vascular smooth muscle cells, and reduce the adhesion of white blood cells and endothelial cells. Ronconi et al. compared the blood of 13 patients with acromegaly and 12 sex- and age-matched normal blood pressure controls and found that the NO concentration decreased in platelets of patients with acromegaly, which was mainly related to the decreased expression of endothelial NO synthase, and the internal NO concentration was negatively correlated with GH/IGF-1 levels and the duration of acromegaly (34).

## Acromegalic Cardiomyopathy

When constantly high levels of GH and IGF-1 stimulated the hearts of patients with acromegaly, and combined with the GH and IGF-1 receptors on the surface of cardiomyocytes, the myocardial contractility changed with increasing the intracellular calcium concentration and sensitivity to calcium, and led to myocardium interstitial collagen precipitation, muscle fiber disorder, and interstitial lymphocyte infiltration of cardiomyocytes, which eventually progressed to acromegaly cardiomyopathy (12, 35). The most common features of acromegaly cardiomyopathy are biventricular concentric hypertrophy, myocardial fibrosis and diastolic dysfunction. In addition, excessive growth hormone may also upset the local water balance of the myocardium and increase the myocardial water content. Excluding other heart diseases, heart changes in acromegaly were first defined as acromegaly cardiomyopathy at the end of the 19th century (36). The development of acromegaly cardiomyopathy is usually divided into three stages. In the early stage, it is mainly myocardial concentric hypertrophy, increasing heart rate and increased cardiac output, which are manifestations of hyperkinetic syndrome generally in younger patients with a shorter duration of disease. Acromegaly cardiomyopathy at this stage is still reversible. In the middle stage, myocardial hypertrophy becomes more obvious and severe, ventricular diastolic function is impaired, and the left ventricular ejection fraction decreases during exercise. If acromegaly is untreated or not effectively controlled, it will progress to the final stage, during which systolic dysfunction at rest and heart failure may occur. At this stage, even if acromegaly is treated, heart disease becomes irreversible (37, 38). In addition, patients with acromegaly often have hypertension, valvular heart disease, arrhythmia, as well as vascular endothelial dysfunction and glucose and lipid metabolism disorders, which may potentially aggravate cardiomyopathy.

### Cardiomyopathy

In 2004, Colao et al. from Italy reviewed the systemic complications of acromegaly and proposed that the most common cardiac change in patients with acromegaly is biventricular concentric hypertrophy. Most patients showed obvious left ventricular hypertrophy at the time of diagnosis. Left ventricular hypertrophy is observed in the early stage of acromegaly, and the histology of acromegaly is mainly manifested as myocardial interstitial fibrosis (12). An autopsy report of 27 patients with acromegaly showed that 93% of patients had left ventricular hypertrophy (LVH), and 85% had myocardial fibrosis (14). At present, most studies exploring changes in cardiac structure and function are conducted through echocardiography (ECHO). **Table 1** summarizes related research on the evaluation of cardiac structure and function in acromegaly with echocardiography from 2002 to 2020 in PubMed. In general, the incidence of myocardial hypertrophy in patients with acromegaly is 10.8-78.9%, and the average incidence is 41.9% (16, 39–59) (**Table 1**). The estimation of the incidence of myocardial hypertrophy is quite different, which may be related to the different definitions of myocardial hypertrophy and the different research designs (retrospective, prospective or cross-sectional) and the different study populations in different studies.

**Table 1:** Echocardiographic assessment of changes in cardiac structure and function in patients with acromegaly from 2002 to 2019.

Author, Published year	Country	Case Number	Sex (M, %)	Age (years)	Duration of illness (years)	GH (µg/l)	IGF-1 (µg/l)	Myocardial hypertrophy (%)	Impaired diastolic function (%)	Impaired systolic function (%)
Lombardi et al., 2002 (39)	Italy	19	9 (47.4)	41.7 ± 11.5	N/A	37.6 ± 47.9	865.7 ± 75.9	78.9	N/A	0
Bogazzi et al., 2005 (40)	Italy	22	13 (59.1)	50.1 ± 10.3	13.1 ± 5.6	14.1 ± 16.7	695.9 ± 291.5	77.2	100%	N/A
Casini et al., 2006 (41)	Brazil	40	14 (35.0)	45 ± 11	8.4 ± 5.1	7.42	2.21 ± 1.28^1^	57.5	30.0	5
Pivonello et al., 2007 (42)	Italy	17	8 (47.1)	46.4 ± 11.1	1.3 ± 0.4	19.8 ± 22.7	731.2 ± 175.8	52.9	58.8	11.8
Bogazzi et al., 2008 (43)	Italy	14	8 (57.14)	46 ± 10	7 ± 5	6.4 ± 9.9	724.8 ± 212	35.7	28.6	0
De Marinis et al., 2008 (44)	Italy	48	18 (37.5)	N/A	N/A	N/A	N/A	70.8	75.0	N/A
Colao et al., 2011 (45)	Italy	205	97 (47.32)	47 ± 15	9.3 ± 6.4	52.1 ± 40.3	2.5 ± 0.9^1^	78.0	43.9	26.3
Jayasena et al., 2011 (46)	UK	116	62 (53.45)	44 ± 2	N/A	N/A	N/A	20	N/A	N/A
Akdeniz et al., 2012 (47)	Turkey	42	15 (35.71)	50.3 ± 12.7	3.8(4.3, 8.9)^2^	N/A	454.7 ± 390.2	33.3	35.7	0
Annamalai et al., 2013 (48)	UK	24	N/A	N/A	N/A	N/A	N/A	58.3	N/A	N/A
Nascimento et al., 2013 (49)	Brazil	37	12 (35.14)	46.9 ± 12.8	N/A	N/A	2.06 ± 0.28^1^	56.8	51.4	0
Mercado et al., 2014 (50)	Mexico	167	N/A	N/A	N/A	N/A	N/A	26.0	N/A	2.4
dos Santos Silva et al., 2015 (51)	Brazil	29	N/A	N/A	N/A	N/A	N/A	31.0	41.3	0
Sanchez-Ortiga et al., 2015 (52)	Spain	32	14 (43.8)	50.3 ± 11.4	6.2 ± 4.8	N/A	N/A	25.0	58.1	N/A
Guo et al., 2015 (53)	China	108	44 (40.74)	41.2 ± 12.7	6.5 ± 5.3	27.4 ± 44.6	802.7 ± 337.5	17.6	20.4	N/A
Kuhn et al., 2015 (54)	France	35	N/A	N/A	N/A	N/A	N/A	20	N/A	N/A
Petrossians et al., 2017 (55)	Europe	3173	1444 (45.5)	45.2(34.9,55)	N/A	N/A	N/A	15.5	N/A	1.6
Cansu et al., 2017 (56)	Turkey	53	21 (39.62)	45 ± 11	6.7 ± 5.9	2.16 ± 3.06^3^	285 ± 204	50.1	11.3	N/A
Carmichael et al., 2017 (57)	US	121	54 (44.63)	55.4 ± 16.7	N/A	N/A	N/A	10.8	N/A	5
Maione et al., 2017 (16)	France	85	N/A	N/A	N/A	N/A	N/A	42.4	N/A	N/A
Natchev et al., 2019 (58)	Bulgaria	146	56 (38.37)	50.6 ± 12.5	N/A	N/A	N/A	48.9	47.0	5.4
Guo et al., 2020 (59)	China	61	34 (55.7)	43.2 ± 13.3	6.5 ± 4.8	36.3 ± 43.7	896.4 ± 253.9	14.8	N/A	3.3

N/A, means not mentioned in the study; %, means the incidence rate; ^1^IGF-1 means a multiple of the upper limit of normal for each age group; ^2^median disease duration; ^3^the unit is ng/ml.

In recent years, studies have found that early pathologies such as reduced myocardial perfusion, myocardial edema, myocardial fibrosis, and regional myocardial movement incoordination greatly affect the quality of life and long-term survival rate of patients, and the application of echocardiography cannot make an accurate assessment (45). Cardiac magnetic resonance imaging (CMRI) has been regarded as the gold standard for the assessment of cardiac lesions in acromegaly (60, 61). At present, there are few studies using cardiac MRI to study acromegaly cardiomyopathy (43, 51, 59, 62–64), and the conclusions are still inconsistent. Bogazzi et al. included 14 patients with acromegaly. CMRI found that 73% of the patients had left ventricular hypertrophy, which was higher than the 36% observed using echocardiography in the same study population. But myocardial fibrosis had not been found in their study (62). However, dos santos Silva et al. (51) included 40 patients and used both CMRI and ECHO to evaluate cardiac structure and function in acromegaly. At baseline, CMRI only found that 5% of patients with acromegaly had LVH, and in delayed myocardial enhancement analysis, 13.5% of patients had myocardial fibrosis, while ECHO found that 31% of patients had LVH. Therefore, the study believed that echocardiography might overestimate the incidence of myocardial hypertrophy. And recently Guo et al. analyzed the CMRI results of 61 patients with acromegaly, found that the incidence of LVH, IVSH, LVSD, RVSD, and myocardial fibrosis were 26.2%, 27.9%, 8.2%, 9.8%, and 14.8%, respectively (59). The main reason for the inconsistency in the diagnosis rate of LVH between the two research centers was the different diagnostic criteria. Different countries and races have dissimilar diagnostic criteria. The study by dos santos Silva et al. defined LVH as left ventricular mass index (LVMi) in males>135 g/m2 and females>110 g/m2, while Bogazzi et al. defined as males>86 g/m2 and females>67 g/m2. The diagnostic criteria of LVH were not mentioned in the report of Guo et al. However, the average LVMi in the study by Guo et al. was very close to the results of studies originating from Brazil, but much lower than the result of European studies (59).

The risk factors for myocardial hypertrophy in patients with acromegaly are still controversial. A retrospective study analyzed the cardiovascular-related complications of 205 newly diagnosed patients with active acromegaly and 410 gender- and age-matched nonacromegalic patients. The results showed that the prevalence of LVH in patients with acromegaly was 11.9-fold higher than that of nonacromegalic patients. The duration of acromegaly was the main risk factor for cardiomyopathy. And the relative risk of cardiomyopathy in patients with a duration of 10 years was 3-fold higher than that of patients with a duration of 5 years (45). A study by Xiaopeng Guo and others from Peking Union Medical College Hospital included 108 patients with acromegaly and found that age and increased BMI were independent risk factors for acromegaly cardiomyopathy (53). In addition, multivariate analysis, such as that performed by Casini, showed that hypertension and IGF-1 levels were determinants of left ventricular hypertrophy (41). However, multivariate logistic regression analysis by Nascimento et al. showed that acromegaly uncontrolled as an independent risk factor for left ventricular hypertrophy (49).

### Heart Function

Increased GH, directly and *via* IGF-1, induces myocardial hypertrophy and fibrosis. LVH causes diastolic and more rarely systolic dysfunction. Diastolic dysfunction is common in patients with acromegaly. According to previous studies, its incidence was approximately 11.3-100%, with an average incidence of 46.3% (40–45, 47, 49, 51, 53, 56, 58) (**Table 1**). Decreased diastolic function is mainly manifested as insufficient cardiac filling capacity, significantly prolonged isovolumic relaxation time (IVRT) and mitral deceleration time (MDT), reduced peak E of mitral valve anterior blood flow, increased peak A of mitral valve anterior blood flow, and reduced E/A ratio, but these situations are usually mild (56). Age and the coexistence of diabetes (diabetics accounted for 19% of patients in this group) are independent risk factors for diastolic dysfunction, regardless of the duration of the disease. Another prospective study involving 19 cases of acromegaly also suggested that age was the most important predictor of diastolic dysfunction (39).

In a national study in Denmark, a total of 405 patients with acromegaly and 4,050 age- and gender-matched healthy subjects were included. The average follow-up was 10.6 years. It was found that the risk of acromegaly heart failure was 2.5 times that of the general population (1). In most studies, impaired systolic function was rare in patients with acromegaly. A comprehensive study of 3,173 patients with acromegaly from the database of 14 medical centers in Europe showed that 1.6% of patients had heart failure when they were diagnosed with acromegaly (55). To date, studies have reported that the highest incidence of impaired systolic function was 26.3%. A total of 205 patients with acromegaly were included in the study. Systolic dysfunction was defined as an echocardiographic examination of cardiac ejection fraction less than 50% (45), but the authors did not clarify whether the patient has clinical manifestations of heart failure. Recently, Guo et al. analyzed the CMRI results of 61 patients with acromegaly. And LVSD was detected in 5 patients (8.2%), 3 of whom had an LVEF higher than 40%. Two patients were diagnosed with severe LVSD and exhibited remarkably decreased LVEF (13.7% and 21.3%). Interestingly, all 5 patients with LVSD were males (59). However, earlier studies used CMRI to evaluate cardiac function did not find patients with acromegaly combined with systolic dysfunction (43, 51, 62). This may be related to the lack of data on the hearts of patients with acromegaly using CMRI assessment. The actual incidence of heart failure of acromegaly in the clinic may be very low. This is due to the continuous improvement in the diagnosis and treatment of acromegaly, which reduces the probability of its terminal stage. It is worthy of attention by clinicians that once patients develop heart failure, they often face a higher risk of death and increased hospitalization costs. In a retrospective study of 330 patients with acromegaly from two centers in France and Belgium (65), 9 patients (2.7%) developed chronic congestive heart failure, which was stage III-IV when rated by the New York Heart Association diagnostic criteria. Echocardiography showed dilated cardiomyopathy with left ventricular systolic dysfunction, and the left ventricular ejection fraction was less than 45%. Among them, 3 patients underwent heart transplantation due to end-stage heart failure. In addition, it was also suggested that if chronic symptomatic congestive heart failure occurred, the 1-year and 5-year mortality rates were 25% and 37.5%, respectively (65). Therefore, when chronic congestive heart failure develops in patients with acromegaly, their condition and prognosis are often poor.

Since obvious impairment of systolic function is rare, some studies have attempted to evaluate the early damage of the myocardium in patients with acromegaly. At present, there are studies using speckle tracking echocardiography (STE) to study the early changes in the left ventricular systolic function of the heart in patients with acromegaly (66–69). STE can analyze the local and overall systolic deformation capacity of the left ventricle, and its average value reflects the global longitudinal strain (GLS) of the left ventricular systolic function. Impaired GLS has been used as a sign of early and subclinical left ventricular systolic dysfunction (66). GLS is also recognized as the main predictor of heart-related events, and its accuracy is even higher than that of LVEF (66). Popielarz-Grygalewic and colleagues evaluated the GLS of 140 patients with acromegaly, and 65% of the patients in this group were women, and the average age was 50.5 ± 13.8 years old. Compared with age- and sex-matched healthy controls, patients with acromegaly had significant subclinical systolic dysfunction (67). Similarly, Uziębło-Życzkowska et al. recently compared 30 cases of acromegaly with 30 cases of a control group matched by sex, age, and blood pressure and found that the left ventricular systolic function of patients with acromegaly was significantly impaired, the GLS value was significantly reduced, and patients with acromegaly had higher left ventricular weight, larger left atrium anteroposterior diameter and left atrium volume index (69). However, Volschan et al. evaluated the GLS of 37 patients with acromegaly and 48 controls (matched by sex, age, hypertension, and diabetes) and found no differences between patients and controls (66). The different conclusions of these studies may be related to sample selection and sample size. At present, there are few studies on GLS in patients with acromegaly, and the early cardiomyopathy of patients with acromegaly needs further study.

## Heart Valve Disease

Previous studies have shown (67, 70–73) that patients with acromegaly often have heart valve disease, among which the mitral valve and aortic valve are the most commonly involved parts, mainly manifested by valve regurgitation. Heart valve disease is an important part of ventricular insufficiency, but there are few reports of valvular disease of acromegaly that are studied separately (70–72). In 1997, Ohtsuka et al. performed valve replacement on 5 patients with severe valve disease and left heart failure and acromegaly. They found that patients with acromegaly had degeneration of heart valves with mucopolysaccharide deposition, increased ring fragility, and valve leaflet disorder. Therefore, regurgitation and stenosis of the mitral valve and aortic valve occurred (73). The cause of valvular disease in patients with acromegaly is not clear. It may be related to the increased expression of matrix metalloproteinases (MMPs), the synthesis of proteoglycans, the deposition of collagen and mucopolysaccharides, and the abnormal regulation of extracellular matrix. In addition, elevated levels of proinflammatory cytokines in patients with active acromegaly could also increase the gene expression of MMPs (70). MMPs are a group of endogenous Zn^2+^-dependent proteolytic enzymes that can degrade collagen, elastin and proteoglycan; can synthesize collagen and connective tissue that do not have normal structure and function by regulating the formation of matrix elements and the bioactive factors released by the degradation of extracellular matrix; and can participate in the remodeling of heart valve tissue (74). Another factor that may cause aortic regurgitation is dilation of the aortic root, which is reported in up to 26% of patients with acromegaly (75, 76).

Patients with acromegaly are prone to various valve diseases, but most valve diseases are mild (70, 71). Colao et al. (71) compared 42 patients with newly diagnosed acromegaly, 22 patients with successful pituitary tumor resection and remission for at least 1 year, and 64 patients with gender- and age-matched nonacromegaly. Mitral and aortic valve calcification, fibrosis, thickening of the valve leaflets and regurgitation were observed. It was found that 86% of patients in the active disease stage had valve abnormalities, while only 24% of the control group had valve abnormalities. After acromegaly was controlled by treatment, 73% of patients had valve abnormalities, while only 9% of the control group had valve abnormalities. However, the degree of valve disease observed in this group of patients was mild to moderate, which was of little clinical significance. Since the prevalence of mild mitral and tricuspid regurgitation is higher in the general population, while the prevalence of aortic regurgitation is much lower, it is generally believed that pathological mitral and tricuspid regurgitation flow is defined as more than moderate regurgitation, and pathological aortic regurgitation is defined as more than mild. Pereira et al. (70) evaluated the pathological significance of valvular regurgitation in patients with acromegaly. The study included 40 patients with acromegaly and 120 in the control group with age, sex, hypertension, and left ventricular systolic function matching. Valve regurgitation with pathological significance was common in patients with acromegaly, and 30% of whom had aortic valve regurgitation, while the incidence rate in the control group was only 7%. Moderate or above mitral regurgitation occurred in 5% of patients with acromegaly, but it was not found in the control group (70).

The duration of acromegaly is considered to be an independent risk factor for valvular disease (70). Study by Pereira et al. suggested that for every additional year of disease duration, the risk of valvular disease increased by 19%, regardless of the levels of GH and IGF-1, whether there was impaired left ventricular function, or hypertension. There was no pathological regurgitation in patients with a duration of less than 6 years, but aortic regurgitation occurred in 12.5% of patients with a duration of 6-10 years, and aortic regurgitation occurs in 40% of patients with a duration of more than 16 years. Moderate and above mitral regurgitation was not observed in the nonacromegalic control group matched for age, gender, hypertension and left ventricular systolic function, but in patients with acromegaly with a duration of more than 16 years, 20% had moderate or more mitral regurgitation (70). Furthermore, the progression of valve disease is related to the failure to effectively control the levels of GH and IGF-I. A prospective study included 18 patients with active acromegaly and patients with acromegaly who were in remission after treatment. It was found that patients with uncontrolled acromegaly had an increased incidence of mitral regurgitation during follow-up; at baseline, 39% had mitral regurgitation, which increased to 78% after an average of 1.9 years of follow-up. However, there was no increase in the incidence of regurgitation in patients with remission of acromegaly (72). However, few studies have assessed the risk factors for valve diseases, especially prospective studies, and more clinical evidence is needed in the future.

## Arrhythmia

In previous studies, 7-40% of patients with acromegaly might have arrhythmia, especially during exercise (37). Arrhythmias mainly manifest as ectopic beats, paroxysmal atrial fibrillation, paroxysmal supraventricular tachycardia, sick sinus syndrome, ventricular tachycardia and bundle branch block. Malignant ventricular tachyarrhythmia may be the cause of repeated syncope and sudden cardiac death in patients with acromegaly (77). The incidence of premature ventricular contractions and complex ventricular arrhythmia in patients with acromegaly is higher than that of the general population (78). In 1992, a clinical observation study included 32 patients found that 48% of patients with acromegaly had complex ventricular arrhythmias (79). In 2002, Lombardi et al. performed a 24-hour dynamic electrocardiogram on 19 patients with acromegaly, and the results showed that approximately 16.6% of patients with acromegaly had supraventricular premature beats, and 35% of patients had ventricular premature beats (39). However, in recent years, relatively few studies had used Holter to assess arrhythmia in acromegaly (80, 81). Recently, a study from Russia found that 42% patients with acromegaly had arrhythmias and cardiac conduction disorders, and 61% of patients with arrhythmia who underwent CMRI had the signs of myocardial fibrosis (82). But Warszawski et al. observed 36 patients with acromegaly and found no persistent arrhythmia at baseline and 1 year after treatment with somatostatin analogs (SSAs), and no arrhythmia-related symptoms were observed (64). The authors speculated that this phenomenon might be related to the fact that there was no significant myocardial fibrosis or myocardial hypertrophy in this group. Another study conducted 24-hour dynamic ECG monitoring on 47 patients with acromegaly and found that the average heart rate and variability of patients with acromegaly increased, but no significant clinical arrhythmia was observed (83).

One study suggested that the duration of acromegaly was a main risk factor of arrhythmias and cardiac conduction disorders (82). In addition, the occurrence of ventricular arrhythmia may be related to left ventricular hypertrophy and fibrosis (63). Some recent studies suggested that the QT interval prolongation or an increase in QT dispersion (84, 85), the frequency of late potentials (81), may increase the risk of acromegaly arrhythmia. The actual reduction in severe arrhythmia in the clinic may be related to the adoption of stricter diagnostic criteria for acromegaly and early intervention in recent years, as well as to the treatment of complications (mainly hypertension and diabetes) that increase the risk of heart disease (64).

## Atherosclerosis and Coronary Heart Disease

There is still controversy about whether patients with acromegaly are at increased risk of atherosclerosis and coronary heart disease (13). Because acromegaly is prone to being associated with hypertension, diabetes and other metabolic-related diseases, the risk of atherosclerosis and coronary heart disease may increase. However, some studies suggested that IGF-1 has anti-inflammatory and antioxidant properties, so IGF-1 was believed to have a protective effect on atherosclerosis (86). Von der Thusen and colleagues observed the effect of IGF-1 on mouse arterial smooth muscle cells cultured in activated macrophage media. IGF-1 in atherosclerotic plaques could prevent plaque instability by regulating the renewal of smooth muscle cells and changing the phenotype of smooth muscle cells (87). Some studies have shown that patients with acromegaly have not found an increased risk of coronary heart disease (88–91). dos Santos Silva et al. compared the coronary artery calcium scores (CACs) and Framingham risk score (FS) of 56 patients with acromegaly and the control group (matched by sex, age, smoking habits, hypertension, diabetes and hypercholesterolemia). Interestingly, no difference was found. According to the FS and CACs to assess the risk of ischemic events, 91% of patients with acromegaly were classified as low-risk (88). Similarly, the study by Akutsu et al. suggested that CACs in patients with acromegaly were lower, and no cardiovascular events occurred after an average follow-up of 4.6 years (89). A prospective study by Bogazzi et al. used the Agatston score (AS) to evaluate the coronary calcium content of 52 patients with acromegaly and stratified them according to the FS. It was found that 71% of patients had a low risk of coronary heart disease, 27% had moderate risk, and 2% had high risk. In all AS-positive patients with calcified plaques in the coronary arteries, myocardial exercise stress SPECT was used to detect ischemia. Amazingly, all patients were negative. During the 5-year follow-up period, no major cardiovascular events occurred (90). Meanwhile, many evidence supports that acromegaly is associated with an increased risk of coronary artery disease (92–95). A study by Ragonese et al. enrolled 39 patients with acromegaly. According to a comprehensive assessment of the FS and AS, 41% of patients with acromegaly were at risk of coronary atherosclerosis (92). A recent meta-analysis of preclinical markers of atherosclerosis in patients with acromegaly also showed that IMT of patients with acromegaly increased, and that both FMD and arterial stiffness were affected, which indicated that these patients had an increased risk of atherosclerosis. But in patients with biochemical remission, IMT and FMD were significantly improved (93). In addition, Ozkan used early markers of atherosclerosis, common carotid artery intima media thickness (IMT), flow-mediated dilation (FMD), and epicardial adipose tissue thickness (EAT) to detect whether patients with acromegaly had early atherosclerosis. The results showed that compared with the control group (matched by age, sex, BMI, diabetes, hypertension, and hyperlipidemia), patients with acromegaly had significantly higher IMT and EAT, while FMD significantly decreased, However, compared with the control group the patients with acromegaly presented lower levels of highly sensitive C reactive protein (hsCRP) and oxidative stress parameters, which suggested that inflammation and oxidative stress did not seem to contribute to the development of atherosclerosis in these patients (94).

There are still controversies about the duration of acromegaly and whether GH and IGF-1 are related to the increased risk of coronary heart disease. Some previous studies have suggested that the risk of coronary heart disease in patients with acromegaly is mainly related to metabolic complications such as diabetes, hypertension, and hyperlipidemia (89, 90). Tellatin and colleagues recently evaluated coronary flow reserve (CFR) as an indicator of coronary microvascular function in patients with asymptomatic acromegaly. The results suggested that patients with acromegaly had lower CFR. CFR and IGF-1 levels were negatively correlated. In the multiple logistic regression analysis, IGF-1 independently increased the probability of CFR ≤ 2.5 (96). The study by Herrmann et al. suggested that the duration of acromegaly and the subsequent metabolic disorders seemed to affect the CAC degree of patients with acromegaly (97).

However, the incidence of coronary heart disease in patients with acromegaly does not seem to increase. A study by Schöfl et al. observed 479 patients with acromegaly in 7 German endocrine centers and found that the incidence of myocardial infarction in patients with acromegaly was very close to that of the general population (SIR: 0.89, 95% CI: 0.47-1.52), P=0.80) (98). Similarly, a national cohort study in Denmark did not find an increase in myocardial infarction in patients with acromegaly (HR: 1.0, 95% CI: 0.5-1.9) (1). Therefore, more prospective studies are needed on the incidence of atherosclerosis and coronary heart disease in patients with acromegaly.

## The Potential Benefits of Acromegaly Control for Related Heart Disease

At present, surgical removal of tumors is still the preferred treatment for patients with acromegaly. Approximately 50% of patients are controlled by surgical treatment, total tumor resection can be easily achieved for microadenoma, and up to 85% of microadenoma is completely removed by surgery. The experience of the neurosurgeon has a great influence on the success rate of the surgery (10, 99). For patients with high-risk surgery or who refuse surgery, and patients whose tumor is located in the cavernous sinus, which may be difficult to cure through surgery, medications (somatostatin analogs, growth hormone receptor antagonist, and dopamine agonist) and radiation therapy could be considered as treatment options (100). 

A large number of clinical observation studies have shown that the heart structure and function of patients with acromegaly are significantly improved after the condition is controlled. It has been reported that surgical treatment can reduce the mass of the left ventricle and improve the diastolic and systolic function of patients (44, 101). Patients with acromegaly who had biochemical control after surgery showed reduced heart rate and blood pressure and improved endothelial diastolic dysfunction (102, 103). Minniti and colleagues included 30 newly diagnosed patients with acromegaly and completed echocardiography before and 6 months after transsphenoidal surgery. The results showed that LVM, LVMi, interventricular septum diastolic thickness (IVSDT) and posterior wall diastolic thickness (PWDT) were significantly reduced within 6 months after surgery. Furthermore, diastolic function was significantly improved compared with preoperative function (101).

At present, drugs such as somatostatin analogs, GH receptor antagonists and dopamine receptor agonists are often used as adjuvant treatments for surgery to achieve biochemical control in patients with acromegaly (10, 39, 61, 100, 103, 104). Many studies have suggested that somatostatin analogs can benefit the heart in treating acromegaly (39, 61, 104). Lombardi and colleagues enrolled 19 patients. After 6 months of treatment with lanreotide, LVM was significantly reduced, and the proportion of patients with ventricular premature beats decreased by 50%. Holter results showed that the heart rate in 24 hours was significantly reduced after 6 months of lanreotide treatment (39). Bogazzi et al. used CMRI to evaluate heart changes in 14 patients with untreated active acromegaly before and after a 6-month duration of treatment with lanreotide. The authors found that short-term treatment with lanreotide reduced the LVMi, reversing LV hypertrophy in most patients. However, no correlation was found between changes in the LVMi and changes in the serum IGF-I concentration. Notably, patients with controlled disease showed greater reduction of the LVMi than those with uncontrolled acromegaly (62). Octreotide and lanreotide may have a direct beneficial effect on the cardiovascular system through somatostatin receptors on cardiomyocytes. Cardiac fibroblasts express somatostatin receptors (SSTRs) 1, 2, 4, and 5. Cardiomyocytes express SSTR1 and SSTR2, and octreotide and lanreotide can bind to SSTR2 and SSTR5 and have a direct effect on cardiac fibroblasts and cardiomyocytes (105, 106). Thus, regardless of whether the administration of somatostatin in patients with acromegaly achieves biochemical control, it may significantly reduce LVMi and improve cardiomyopathy. Meanwhile, somatostatin analog therapy may improve mortality in patients with acromegaly. In 2019, a meta-analysis evaluating the mortality of acromegaly revealed that in 6 clinical intervention studies that used somatostatin analogs as an adjuvant treatment for patients after surgery, the mortality of acromegaly did not increase (SMR: 0.98, CI: 0.83-1.15), and only in patients who received surgery and/or radiotherapy without the use of somatostatin analogs as adjuvant treatment did the mortality rate significantly increase (SMR: 2.11; CI: 1.54-2.91) (11).

When SSAs treatment alone cannot achieve biochemical control of acromegaly, the SSAs can be replaced or combined with the growth hormone receptor antagonist pegvisomant (PEG) (107). When SSAs fail to control acromegaly, PEG normalized serum IGF1 levels in 70–97% of cases (54). Studies have suggested that long-term SSAs and PEG combined therapy can improve the cardiac structure and function in patients with acromegaly resistant to SSAs, especially patients with diastolic dysfunction (54, 108). Auriemma et al. evaluated 36 patients undergoing high-dose single-drug-resistant SSA therapy. These patients received SSA treatment for a median duration of 36 months and further received SSA+PEG treatment for a median duration of 60 months. Comparison of the cardiac structure and function after using an SSA alone, using an SSA+PEG for 12 months, and using an SSA+PEG for 60 months suggested that short-term and long-term SSA+PEG treatment could significantly improve the LVMi and E/A. The study also suggested that the improvement was mainly related to IGF-1 normalization, and the IGF-1 concentration in 83% patients was controlled to the normal range (108). Because GH receptors are expressed on cardiomyocytes, PEG might have a direct effect on the heart; however, this remains to be elucidated (109). Few studies to date have focused on the changes in cardiac structure or function in patients with acromegaly using PEG alone (42, 54). A study by Pivonello et al. suggested that the LVM decreased after long-term use of PEG in 12 patients with acromegaly, both diastolic and systolic function improved, and a correlation was found between changes in the IGF-I concentration and changes in the left ventricular ejection fraction (42). However, a study by Kuhn et al. indicated that the LVM decreased and that the LVEF did not change significantly overall. Notably, they found that the LVEF significantly improved in patients with a baseline LVEF of <60% but decreased in patients with an LVEF of >70% (54). In addition to improving the structure and function of the heart, PEG can also improve arrhythmias (95) and reduce the Framingham risk score (110).

For patients with acromegaly who have not achieved biochemical remission after surgery, dopamine receptor agonist therapy is also a treatment options, especially for those patients with only a mildly elevated serum IGF-1 concentration. Previous studies have shown that high-dose cabergoline is related to valve disease in patients with Parkinson’s disease (111, 112). A recent meta-analysis showed that patients with hyperprolactinemia treated with cabergoline were at increased risk of regurgitation of the tricuspid valve. This study analyzed data from 13 published studies of 836 patients with hyperprolactinemia treated with cabergoline and 1388 healthy controls. The results suggested that there was no difference in the risk of aortic or mitral regurgitation between the cabergoline-treated patients and the control group (113). To the best of our knowledge, only one study has evaluated the effects of cabergoline on cardiac valves in patients with acromegaly (114). Maione et al. compared the prevalence and incidence of heart valve disease and regurgitation in a series of patients with acromegaly treated with cabergoline and matched patients who had never received this drug. The authors found no increase in the prevalence of valve regurgitation or remodeling relative to a matched cohort of patients with acromegaly who had never received cabergoline. The valve abnormalities observed here are more likely to have been related to acromegaly itself than to cabergoline (114). However, the cumulative doses and treatment durations in patients with acromegaly and patients with hyperprolactinemia were consistently lower than those in patients with Parkinson’s disease. One study suggested that for patients who need high-dose cabergoline (>3 mg/week) for a long period of time, echocardiography may be needed to evaluate valve abnormalities, whereas patients who receive low-dose cabergoline (1–2 mg/week) may not need routine echocardiography (115). However, whether cabergoline might be harmful to the cardiac structure in patients with acromegaly requires further evaluation.

## Summary

In summary, cardiovascular complications are the most common complication in patients with acromegaly, and also the main factors affecting the quality of life and survival time of patients. Patients with acromegaly often have cardiovascular diseases such as hypertension, myocardial hypertrophy, diastolic insufficiency, and arrhythmia. At present, the underlying physiopathology of various cardiac complications has not been fully elucidated, but the coexistence of GH/IGF-I over secretion, older age at diagnosis, prolonged disease duration, and other cardiovascular risk factors may be the most important factors for cardiovascular complications. Once acromegaly is diagnosed, the patient’s heart condition should be thoroughly evaluated immediately. Clinicians should carefully screen each patient for hypertension, cardiomyopathy, valvular heart disease, and arrhythmia. More importantly, active intervention is necessary. Surgery is the first-line choice for patients with acromegaly to remove GH pituitary adenomas and control GH/IGF-1 levels. For patients who have not remitted after surgery and cannot be operated on, radiotherapy and/or drug therapy should be considered to achieve biochemical remission of GH/IGF-1 levels in patients with acromegaly as soon as possible. Early diagnosis and treatment can help reduce the risk of severe cardiac complications in patients with acromegaly, improve the quality of life of patients, and prolong the survival time.

## Author Contributions

HY and HT designed, viewed, and collected the literature, analyzed the data, wrote and revised the draft, and contributed equally to this work. HH analyzed and summarized the research progress of cardiac imaging, JL designed and analyzed, and contributed to the revision of the manuscript. All authors contributed to the article and approved the submitted version.

## Funding

This study was supported by Grant 1.3.5 project for Disciplines of Excellence Clinical Research Incubation Project, West China Hospital Sichuan University (Grant No. 2020HXFH034).

## Conflict of Interest

The authors declare that the research was conducted in the absence of any commercial or financial relationships that could be construed as a potential conflict of interest.
